# Hydrolysis Reactions of p-Nitrophenyl Trifluoroacetate and S-Ethyl Trifluorothioacetate

**DOI:** 10.3390/molecules30020268

**Published:** 2025-01-11

**Authors:** Jack B. Suggs, Joshua J. Melko

**Affiliations:** Department of Chemistry and Biochemistry, University of North Florida, Jacksonville, FL 32224, USA; n01541928@unf.edu

**Keywords:** water structures, kinetic rates, esters, thioesters, potential energy surface, cleavage reactions, hydrogen bonding effects, general solvation effects

## Abstract

The formation of water structures can provide significant benefits in organic reactions, stabilizing charge and lowering activation energies. Hydrolysis reactions will frequently rely on water networks to accomplish these goals. Here, we used computational chemistry and experimental kinetics to investigate a model thioester molecule S-ethyl trifluorothioacetate, and extended work on a previously characterized ester p-nitrophenyl trifluoroacetate. We found that the rate-determining steps in these reactions are heavily influenced by the nature of the leaving group. The hydrolysis of S-ethyl trifluorothioacetate was much slower than p-nitrophenyl trifluoroacetate for this reason. We explored differences in the reaction orders with respect to water and examined details of calculated potential energy surfaces of these hydrolysis reactions, highlighting the roles of solvation effects and transition state structures.

## 1. Introduction

**Figure 1:**
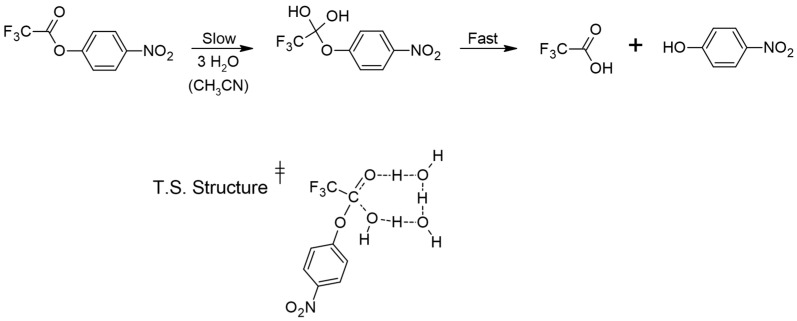
Mechanism of hydrolysis in acetonitrile of p-nitrophenyl trifluoroacetate. The rate-limiting transition state, (^‡^), is shown at the bottom, consisting of an eight-membered ring of three water molecules.

The formation of water networks to catalyze organic reactions has long been known as a method to decrease the enthalpy required to initiate reactions [1]. These processes are relevant in a variety of gas phase and liquid phase chemical systems, including biological, environmental, and atmospheric systems, with special attention paid to solvation effects in liquid systems [2,3]. Molecular reactivity in hydrolysis reactions, where water networks may inevitably be involved, depends on the efficiency of the leaving group, which in turn depends on its basicity [4]. Specifically, hydrolysis of esters typically forms tetrahedral intermediates, which rapidly decompose to products. This rapid decomposition is best understood by examining the leaving groups involved in the decomposition relative to the reversal of the intermediate back to reactants. For example, previous work has examined the water-catalyzed hydrolysis of p-nitrophenyl trifluoroacetate in acetonitrile (i.e., a non-aqueous medium), suggesting that a water bridge of three water molecules hydrates this ester in a rate-limiting addition of water to form a tetrahedral intermediate [4]. This intermediate rapidly expels the leaving group, p-nitrophenoxide, to form the products. This leaving group being a relatively weak base (pK_a_ of the conjugate acid is ≈7.5) justifies the quick expulsion compared to the formation of the intermediate. Thus, kinetic studies of this reaction pertain to the addition step rather than the rapid elimination step. This mechanism is summarized below in Figure 1, where the transition state arranges three water molecules in an eight-membered ring to maintain linear hydrogen bonds in an attempt to become enthalpically favored despite sacrificing entropy.

Recently, the hydrolysis of esters has gained renewed interest in the fields of origins of life and biochemistry. Goldford et al. suggested that thioester-based metabolism could pre-date the incorporation of phosphate in our RNA-based genetics [5]. Sanden et al. have combined experimental reaction rates with density functional calculations in a polarizable solvent continuum to explore the origin of metabolism in the specific context of the hydrolysis of thioesters, finding that high-temperature acidic conditions are favored [6]. In biochemical applications, many studies explore tetrahedral intermediate formation when enzymes hydrolyze esters. Aranda et al. performed calculations to reveal catalytic mechanisms of carboxylesterases, a class of enzymes that cleave carboxyesters via proton transfer hydrolysis involving multiple tetrahedral intermediates [7]. This action is involved in the metabolism of clinical drugs, as well as cholesterol and fatty acid metabolism. Finally, Patel et al. have undertaken QM-cluster and QM/MM studies of thiohemiacetal intermediates with an enzyme whose active site is similar to thioesterases, demonstrating that hydrolysis with a water molecule is not possible in their case [8].

Here, our studies examined the mechanism of the hydrolysis of p-nitrophenyl trifluoroacetate, as shown in Figure 1. We used computational chemistry to calculate the enthalpy of reaction as a function of the number of waters involved. Specifically, we used the Gaussian 09 software at the B3LYP-D3/6-311++G(d,p) level, including empirical dispersion calculations [9,10,11,12]. The B3LYP-D3/6-311++G(d,p) level was chosen through benchmarking against experimental values, and selected due to its superior results compared to other approaches, such as MP2 or 6-31+G(d,p). We studied multiple configurations of p-nitrophenyl trifluoroacetate to explore various reaction pathways, each with 1, 2, 3, 4, 5, and 11 individual water molecules.

**Figure 2:**
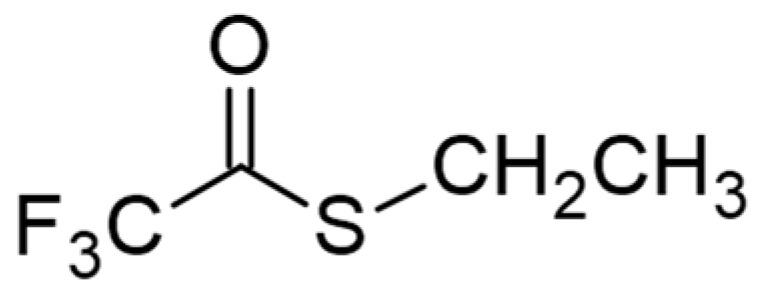
Structure of S-ethyl trifluorothioacetate.

In addition, we studied a second molecule, S-ethyl trifluorothioacetate (Figure 2,), both computationally, with the same functional and basis set for up to five water molecules, and experimentally, with a UV–Vis spectrometer using kinetic rate methods, employing various water molarities in an acetonitrile solvent.

Its mechanism will be similar to that shown for p-nitrophenyl trifluoroacetate in Figure 1, forming a tetrahedral intermediate that rapidly decomposes by expelling the reasonably good leaving group CH_3_CH_2_S^−^ (pK_a_ of the conjugate acid, CH_3_CH_2_SH is ≈10.5). Since this leaving group is not quite as good as that of p-nitrophenyl trifluoroacetate, we expect that the thioester here will react more slowly. Water networks catalyze a two-step addition–cleavage reaction in p-nitrophenyl trifluoroacetate through the hydration and reformation of a carbonyl, and a similar two-step addition-cleavage reaction in S-ethyl trifluorothioacetate is expected. We found differences in reaction orders with respect to water and we have discussed the reaction mechanisms found from calculated potential energy surfaces. This has allowed us to expand on previous results for p-nitrophenyl trifluoroacetate and shine light on the role of general solvation effects and hydrogen bonding in hydrolysis reactions of similar molecules.

## 2. Results and Discussion

Our experimental rate constants for S-ethyl trifluorothioacetate are shown below in Table 1. As hypothesized, the rates at 23.0 °C are much slower than the comparable ones for p-nitrophenyl trifluoroacetate measured previously, 0.0000370 s^−1^ for S-ethyl trifluorothioacetate versus 0.000281 s^−1^ for p-nitrophenyl trifluoroacetate in reference [4]. Rate constants measured here were between 3.7 × 10^−5^ at their lowest, and 3.4 × 10^−4^ at their highest. At 23.0 °C, the 5 M H_2_O rate constant was about 1.0 × 10^−4^ s^−1^. This reaction has been measured in pure water previously, where at a rate of 25.0 °C, constants were about 5.0 × 10^−2^ s^−1^ [13].

As shown in Table 1, our experimental results for the hydrolysis of S-ethyl trifluorothioacetate in acetonitrile were obtained at a variety of water concentrations and multiple temperatures. By keeping the concentration of S-ethyl trifluorothioacetate much lower than water, we are able to obtain pseudo-first-order rate constants from our experiments. These observed rate constants (k_observed_ or k_obs_) represent the true rate constant multiplied by the water concentration raised to the power of x, where x represents the order with respect to water:

(1) $$k_{observed}=k{\left[H_{2}O\right]}^{x}$$

This allows us to plot a series of observed rate constants versus water concentrations to yield a linear relationship, with a slope giving the apparent order with respect to water:

(2) $$\log k_{observed}=\log k+x\log\left[H_{2}O\right]$$

**Figure 3:**
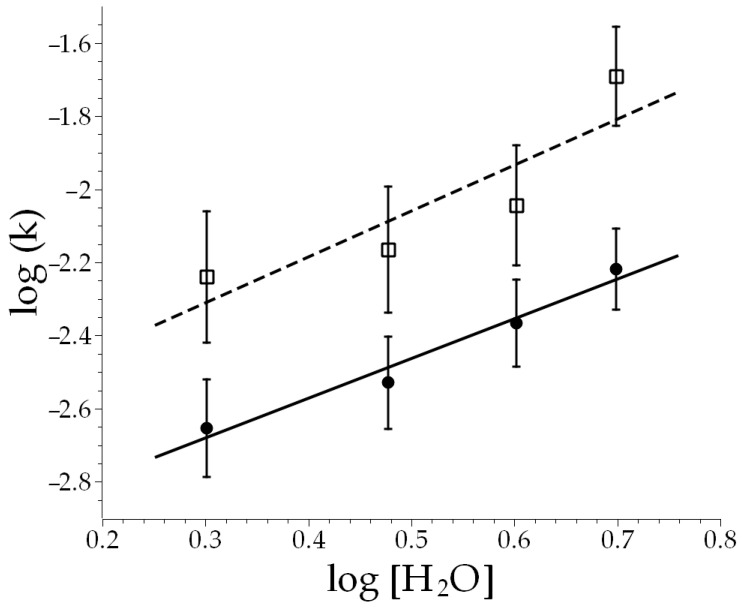
A plot of observed rate constants versus water concentration (in M) for the first step of the hydrolysis reaction of S-ethyl trifluorothioacetate. The black dots and the solid line represent measurements taken at 23.0 ± 0.1 °C, and the white squares and the dashed line represent measurements taken at 45.0 ± 0.1 °C. Error bars represent standard deviation.

Figure 3 shows the results of such plots. The circles represent the 23.0 °C rate constants, while the open squares represent the 45.0 °C rate constants. The best fit lines are shown for each temperature, and their similar slopes suggest similar orders with respect to water at these two different temperatures, which is expected. It is also evident that the higher temperatures lead to higher rate constants, with the rate constants more than doubling with the raise in temperature. The slopes of the lines in Figure 3 provide the order with respect to water for this reaction, and at both temperatures, we obtained an order of about one (1.09 at 23.0 °C and 1.33 at 47.0 °C).

**Figure 4:**
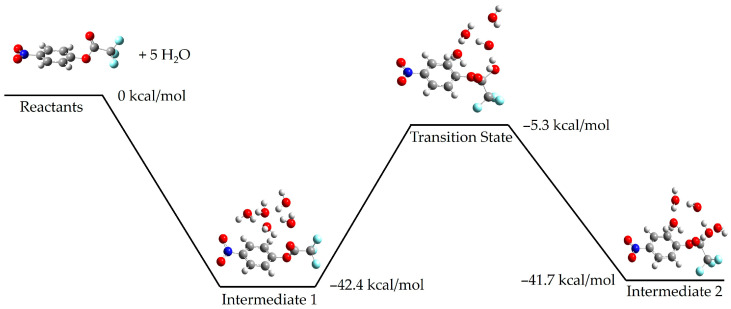
The computationally calculated potential energy surface of p-nitrophenyl trifluoroacetate and five individual water molecules. With a five water ring, the transition state (measured at −5.3 kcal/mol, relative to the reactants) becomes lower than the reactants. Reported values are enthalpies. Carbon atoms are grey, hydrogen atoms are white, oxygen atoms are red, fluorine atoms are light blue, and the nitrogen atom is dark blue.

**Figure 5:**
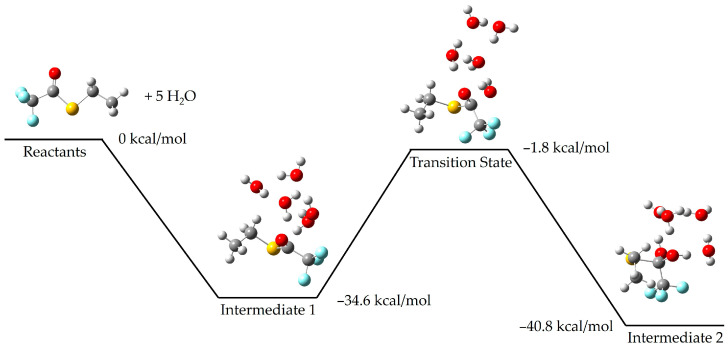
The computationally calculated potential energy surface of S-ethyl trifluorothioacetate and five individual water molecules. With a five water ring, the transition state (measured at −1.8 kcal/mol, relative to the reactants) becomes lower than the reactants. Reported values are enthalpies. Carbon atoms are grey, hydrogen atoms are white, oxygen atoms are red, fluorine atoms are light blue, and the sulfur atom is yellow.

To understand more details of the mechanisms at play here, we have conducted calculations of the reaction coordinates for the initial step shown in Figure 1, identifying the transition state structure and barrier heights involved for the reactions of both p-nitrophenyl trifluoroacetate and S-ethyl trifluorothioacetate with a series of water molecules (*n* = 1–5). For both p-nitrophenyl trifluoroacetate and S-ethyl trifluorothioacetate, we found that a structure incorporating five waters is required before the reaction is thermodynamically allowed, with these waters typically arranged in a ring structure. The potential energy surfaces for p-nitrophenyl trifluoroacetate and S-ethyl trifluorothioacetate reacting with five water molecules are shown below, in Figure 4 and Figure 5, respectively. The transition state barrier becomes −1.8 kcal/mol below the reactants for S-ethyl trifluorothioacetate, while for p-nitrophenyl trifluoroacetate, the corresponding value is −5.3 kcal/mol. Calculations with fewer water molecules (e.g., *n* = 1–4) possessed barriers that were endothermic. However, we should note that this does not indicate that the order with respect to water is five, but rather that this number of water molecules is necessary to meet both the required order with respect to water as well as sufficient solvation effects, to bring the barrier down below reactant energies and allow for this reaction at room temperature. These surfaces are still consistent with our experimental results, but supplement them with a qualitative picture of the reaction coordinate involved.

**Figure 6:**
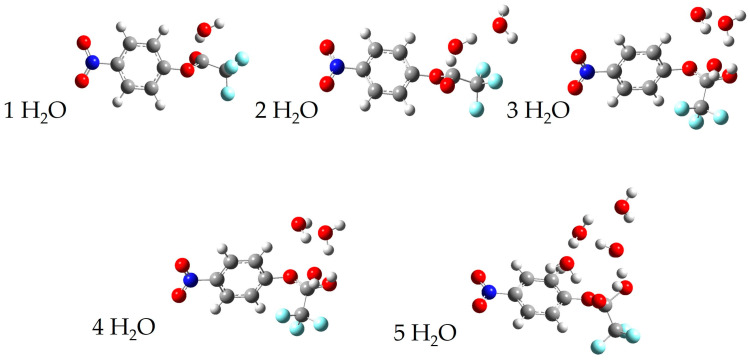
A depiction of the transition states of p-nitrophenyl trifluoroacetate with one, two, three, four, and five individual water molecules.

**Figure 7:**
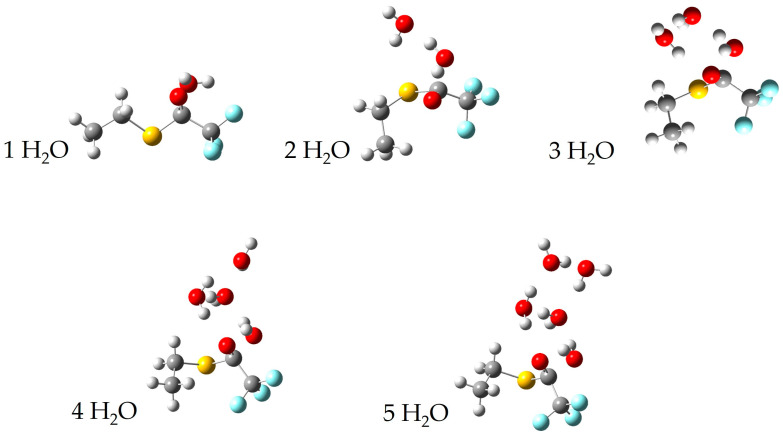
A depiction of the transition states of S-ethyl trifluorothioacetate and one, two, three, four, and five individual water molecules.

We have calculated surfaces similar to the figures above for each species with 1–4 water molecules, as well as 11 water molecules in the case of p-nitrophenyl trifluoroacetate. The full cartesian coordinates and energies of stationary points can be found in the Supplementary Materials. For brevity, we only show below the calculated transition state for each series of water molecules with p-nitrophenyl trifluoroacetate and S-ethyl trifluorothioacetate in Figure 6 and Figure 7, respectively. The previous literature on the topic of p-nitrophenyl trifluoroacetate hydrolysis experimentally reports an order of about three with respect to water [4]. Our experimental results determined here for the hydrolysis of S-ethyl trifluorothioacetate found an order of about one with respect to water. Our calculated surfaces and transition state structures complement both of these experimental results, providing a picture of the mechanisms at play and elucidating the extent of solvating waters necessary to make these reactions thermally accessible at our reaction conditions. We note that in S-ethyl trifluorothioacetate’s hydrolysis reaction individual water molecules tend to form a more independent ring structure, only loosely communicating with the water molecule performing the hydrolysis. This is in contrast to p-nitrophenyl trifluoroacetate’s hydrolysis reaction, in which individual water molecules tend to be more rigidly involved with the water molecule performing the hydrolysis reaction, as well as coordinating to the organic molecule. Polar and hydrogen-bonding solvents tend to affect reaction dynamics due to solvation effects, and in the liquid phase hydrogen-bonding molecules can form extensive networks to allow for greater reaction stability and thus a decrease in activation energy compared to the gas phase [2,3]. Thus, we believe our experimental results and the previous literature results identify the order with respect to water in these hydrolysis reactions, while our computational results provide a qualitative look at the structures and energetics involved in an idealized gas phase-type environment. For S-ethyl trifluorothioacetate’s hydrolysis only one water molecule is involved in the rate-limiting step, with the waters beyond that providing beneficial solvation effects while not being included in the order of the reaction. The previous literature on the topic of p-nitrophenyl trifluoroacetate suggested an order of about three to be the most reasonable, but suggested that large numbers of water molecules may be involved in the rate-determining step as well [4]. The suggested three water molecules are what is shown in Figure 1 for that reaction, whereas our computational methods are probing a more isolated reaction with far fewer total water molecules, but providing a more detailed picture (e.g., the top-right of Figure 6) of the same three water molecule transition states shown in Figure 1. The precise energetics of hydrolysis of these molecules is significantly affected by general solvation and longer hydrogen bonding networks, but the computational results show their reactions are thermodynamically allowed once, only accounting for five total water molecules. Comparing the two potential energy surfaces in Figure 4 and Figure 5, one can see that both are thermodynamically accessible relative to the reactants, with the thioester’s transition state being less submerged (i.e., −1.8 kcal/mol relative to reactants) than the nitrophenyl’s transition state (i.e., −5.3 kcal/mol relative to reactants). While one might suggest that this justifies the experimentally observed slower reaction rate for the thioester, we believe that these calculations offer only a qualitative picture, and in fact one could make an argument that the transition state barrier relative to the intermediate energy is the more important value, which actually favors the thioester.

## 3. Materials and Methods

Experiments were performed using a ThermoFisher Evolution 220 UV–Vis Spectrometer (Thermo Fisher Scientific, Waltham, MA, USA), set to a wavelength of 224 nm. A Beckman fused quartz cuvette was used for the reaction, containing 3 mL of 2 M, 3 M, 4 M, or 5 M deionized H_2_O solution in acetonitrile (Sigma Aldrich, St. Louis, MO, USA, ≥99.9%), with 893 μL of HCl (Fisher Scientific, Waltham, MA, USA, ACS Grade). The cuvettes were either held in the stock Thermo Fisher cuvette holder, or with a custom temperature controlled 10-mm cell holder purchased from Thermo Scientific, equipped with special tubing to allow for the circulation of heated water external to the cuvette. Cuvettes, once up to temperature, were then spiked with 100 μL of 0.005 M S-ethyl trifluorothioacetate in acetonitrile and inverted to ensure proper mixture. Kinetic reactions were monitored at temperatures of 23.0 °C ± 0.1 or 47.0 °C ± 0.1. Kinetic data were then exported to analysis software and reviewed using first-order rate constants determined from the plots of log(A_t_ − A_∞_).

Geometries and reaction coordinates were calculated using the Gaussian 09 software package (Gaussian Inc., Wallingford, CT, USA) [9,10,11]. A variety of functionals, including B3LYP and MP2, were explored, along with basis sets (e.g., 6–311, 6–31), before settling on B3LYP-D3/6-311++G(d,p) due to the consistency and validity of the results versus established reactions, as well as its balanced use of available computational resources [9,10,11,12,14,15,16,17,18,19]. The established reactions used were the ionization of 1-acetoxyl-4-fluorobenzene and the ionization of trifluoroacetic acid, which were selected due to the availability of established data and their similarity to our target molecules [20,21]. Both reactions were calculated at about a 2% error computationally compared to the experimental results. To ensure maximum accuracy, empirical dispersion was included using the D3 version of Grimme’s dispersion with Becke–Johnson damping (GD3BJ) for all reaction calculations [12]. This functional and basis set package was used to compute both the p-nitrophenyl reaction pathways and energies and the thioester reaction pathways and energies. Computational work proceeded through optimization and frequency calculations for minima on both sides of a reaction, followed by QST3 calculations to find the ideal transition state, and then intrinsic reaction coordinate calculations to confirm the connection between the transition state and minima on the potential energy surface. Stationary points identified were subjected to frequency calculations, confirming that the minima contained no imaginary frequencies and that transition states contained one imaginary frequency. All reported energies include zero-point corrections. We were additionally particularly careful to analyze a multitude of possible spatial configurations for each molecule, to ensure we found the lowest energy reaction pathway. Enthalpy results for each calculation were then compiled and compared to construct the potential energy surface.

## 4. Conclusions

Water structures can be effective catalytic structures in the hydrolysis of esters. Here, we examined the role of water in hydrolysis reactions of two different organic molecules, p-nitrophenyl trifluoroacetate and S-ethyl trifluorothioacetate. We found very slow reaction rates for S-ethyl trifluorothioacetate compared to the p-nitrophenyl trifluoroacetate and explained the difference in terms of the leaving groups involved. We also assigned an order with respect to water in the case of S-ethyl trifluorothioacetate and compared that to previous experimental work. Our calculated reaction surfaces provide mechanistic details of these hydrolysis reactions and show the ring structures of waters involved that maintain hydrogen bond networks, even in these few-water systems.

We believe this work furthers the understanding of the role of hydrogen bonding and solvation effects in the energetics and mechanisms involved, and we hope to extend this work to more molecules. One viable future research direction would include experiments and calculations on the molecule p-nitrophenyl trifluoro-N-methyl acetanilide. We hypothesize that, in this molecule, the rate-limiting step is actually the expulsion of the leaving group due to its strong basicity, allowing for the further elucidation of the mechanics of ester hydrolysis. Another future research direction could extend these studies to esters where water coordination should be more difficult, such as esters that possess hydrophobic side chains.

## Figures and Tables

**Table 1:** First-order rate constants for the hydrolysis of S-ethyl trifluorothioacetate in mixtures of water–acetonitrile at 23.0 ± 0.1 °C and 45.0 ± 0.1 °C.

Molarity of H_2_O	23.0 ± 0.1 °C 10^4^ k_obs_, s^−1^	45.0 ± 0.1 °C 10^4^ k_obs_, s^−1^
2.0 M H_2_O	0.370	0.900
3.0 M H_2_O	0.493	1.140
4.0 M H_2_O	0.718	1.510
5.0 M H_2_O	1.010	3.400

## Data Availability

The original contributions presented in this study are included in the article. Further inquiries can be directed to the corresponding author.

## Supplementary Materials

The following supporting information can be downloaded at: https://www.mdpi.com/article/10.3390/molecules30020268/s1, Table S1: Energies corresponding to the potential energy surfaces of p-Nitrophenyl Trifluoroacetate reacting with the series of H_2_O molecules studied; Table S2: Energies corresponding to the potential energy surfaces of S-ethyl Trifluorothioacetate reacting with the series of H_2_O molecules studied; Table S3: Cartesian coordinates of each of the intermediates and transition states studied for p-nitrophenyl trifluoroacetate; Table S4: Cartesian coordinates of each of the intermediates and transition states studied for S-ethyl trifluorothioacetate.
