# Supplementary material for: Hydrolysis Reactions of p-Nitrophenyl Trifluoroacetate and S-Ethyl Trifluorothioacetate
Source: Molecules. 2025 Jan 11;30(2):268. doi: 10.3390/molecules30020268 (PMC11767969; doi:10.3390/molecules30020268)
Supplement: Supplementary file 1 [file molecules-30-00268-s001.zip › molecules-3366120-supplementary.pdf]

Supplementary Material for "*Hydrolysis Reactions of p-Nitrophenyl Trifluoroacetate and S-ethyl Trifluorothioacetate*"

Jack B. Suggs II[1] and Joshua J. Melko[1]*

[1]*Department of Chemistry and Biochemistry, University of North Florida*

*Corresponding author: Email: josh.melko@unf.edu

Table S1. Energies corresponding to the potential energy surfaces of p-Nitrophenyl Trifluoroacetate reacting with the series of H_2O molecules studied.

***Presented in the paper are these energies relative to p-Nitrophenyl Trifluoroacetate and the corresponding number of H_2O molecules infinitely separated in space (p-Nitrophenyl Trifluoroacetate energy: -603991.7123526 kcal/mol, H_2O energy: -47963.1078644 kcal/mol.).**

kcal/mol

# of H_2O Molecules	Intermediate 1	Transition State	Intermediate 2
**1**	-651960.244	-651918.394	-651956.014
**2**	-699930.710	-699890.902	-699928.688
**3**	-747896.927	-747853.522	-747891.479
**4**	-795866.118	-795843.580	-795873.786
**5**	-843849.680	-843812.646	-843849.065
**11**	-1131693.049	-1131657.751	-1131692.430

Table S2. Energies corresponding to the potential energy surfaces of S-ethyl Trifluorothioacetate reacting with the series of H_2O molecules studied.

***Presented in the paper are these energies relative to S-ethyl trifluorothioacetate and the corresponding number of H_2O molecules infinitely separated in space (S-ethyl Trifluorothioacetate energy: -582637.8324589 kcal/mol, H_2O: -47963.1078644 kcal/mol).**

	kcal/mol		
**# of H_2O Molecules**	**Intermediate 1**	**Transition State**	**Intermediate 2**
**1**	-630606.027	-630561.660	-630594.546
**2**	-678577.619	-678535.969	-678570.302
**3**	-726546.781	-726510.473	-726544.488
**4**	-774525.520	-774479.788	-774516.897
**5**	-822488.715	-822455.235	-822494.153

Table S3. Cartesian coordinates of each of the intermediates and transition states studied for p-nitrophenyl trifluoroacetate. Naming follows the convention in Figures 4 and 5 in the manuscript.

<u>p-nitrophenyl trifluoroacetate</u>

1 water Intermediate 1

Center Number	Atomic Number	Atomic Type	Coordinates (Angstroms) X	Y	Z
1	6	0	2.207179	1.065525	0.078920
2	6	0	3.005285	-0.073815	0.050145
3	6	0	2.475807	-1.354940	0.155016
4	6	0	1.100331	-1.499096	0.288649
5	6	0	0.306975	-0.360753	0.314437
6	6	0	0.833072	0.921624	0.216460
7	1	0	2.665225	2.040849	-0.002394
8	1	0	3.134357	-2.211165	0.129783
9	1	0	0.643299	-2.476160	0.373768
10	1	0	0.170215	1.776007	0.251076
11	7	0	4.467189	0.082326	-0.093589
12	8	0	4.909692	1.219944	-0.186233
13	8	0	5.147274	-0.935574	-0.110994
14	8	0	-1.067583	-0.533504	0.516384
15	6	0	-1.905772	-0.176771	-0.470855
16	8	0	-1.622925	0.184273	-1.570628
17	6	0	-3.365996	-0.404446	-0.005982
18	9	0	-3.688703	-1.700707	-0.196293
19	9	0	-4.203762	0.343781	-0.730024
20	9	0	-3.552755	-0.118755	1.289127
21	8	0	-2.086330	2.463635	0.372106
22	1	0	-2.583527	2.808981	1.120773
23	1	0	-2.475217	2.872175	-0.408992

1 water Transition state

Center Number	Atomic Number	Atomic Type	Coordinates (Angstroms) X	Y	Z
1	6	0	2.371150	-1.306908	-0.051622
2	6	0	2.991498	-0.068890	0.073091
3	6	0	2.282156	1.092766	0.358145
4	6	0	0.905478	1.015486	0.526448
5	6	0	0.287795	-0.221701	0.393664
6	6	0	0.993819	-1.382195	0.111481
7	1	0	2.962587	-2.183273	-0.274574
8	1	0	2.808579	2.032171	0.447921

9	1	0	0.313170	1.892249	0.748531
10	1	0	0.466753	-2.322399	0.019236
11	7	0	4.456416	0.015018	-0.102141
12	8	0	5.058418	-1.023185	-0.342478
13	8	0	4.977445	1.117454	0.004140
14	8	0	-1.089583	-0.342530	0.624379
15	6	0	-1.936150	0.157161	-0.282729
16	8	0	-1.602128	0.311338	-1.519540
17	6	0	-3.363978	-0.327263	0.073697
18	1	0	-1.807482	1.492007	-1.267381
19	9	0	-3.654498	-0.135923	1.367929
20	9	0	-3.468105	-1.635179	-0.199310
21	9	0	-4.277248	0.330337	-0.659194
22	1	0	-2.925400	2.294399	0.041755
23	8	0	-2.044786	1.925158	-0.116298

1 water Intermediate 2

Center Number	Atomic Number	Atomic Type	Coordinates (Angstroms) X	Y	Z
1	6	0	-2.108163	1.042361	-0.764432
2	6	0	-2.689779	-0.034573	-0.104419
3	6	0	-1.971509	-1.186016	0.199036
4	6	0	-0.636057	-1.270340	-0.170541
5	6	0	-0.042958	-0.188522	-0.821340
6	6	0	-0.770316	0.961425	-1.121847
7	1	0	-2.699020	1.920837	-0.981069
8	1	0	-2.463091	-2.001535	0.710175
9	1	0	-0.057445	-2.152597	0.056114
10	1	0	-0.276172	1.780223	-1.627760
11	7	0	-4.109984	0.046244	0.282103
12	8	0	-4.718446	1.070962	-0.001388
13	8	0	-4.597135	-0.915972	0.862761
14	8	0	1.266732	-0.217943	-1.276505
15	6	0	2.440952	0.552095	0.725443
16	9	0	2.434505	1.792056	0.193924
17	9	0	3.568804	0.406009	1.426008
18	9	0	1.403102	0.459351	1.571207
19	6	0	2.361353	-0.507039	-0.421018
20	8	0	2.313740	-1.743227	0.176277
21	1	0	2.765337	-2.363850	-0.411073
22	8	0	3.486351	-0.428061	-1.218880
23	1	0	3.331519	0.234046	-1.904776

2 water Intermediate 1

Center Number	Atomic Number	Atomic Type	Coordinates (Angstroms) X	Y	Z
1	6	0	-2.810394	-1.117467	-0.743758
2	6	0	-3.267668	0.022347	-0.092081
3	6	0	-2.401850	0.974845	0.436566
4	6	0	-1.032069	0.785071	0.310308
5	6	0	-0.579676	-0.358497	-0.336304
6	6	0	-1.439907	-1.310422	-0.865929
7	1	0	-3.519720	-1.830029	-1.139380
8	1	0	-2.804744	1.846459	0.932213
9	1	0	-0.313636	1.498158	0.693310
10	1	0	-1.037757	-2.182959	-1.363740
11	7	0	-4.723987	0.229227	0.040412
12	8	0	-5.464201	-0.617472	-0.444247
13	8	0	-5.103447	1.235097	0.626019
14	8	0	0.793846	-0.546565	-0.534397
15	6	0	1.569149	-0.747808	0.544771
16	8	0	1.224188	-0.907984	1.672970
17	6	0	3.043805	-0.900600	0.096125
18	9	0	3.340218	-0.191465	-1.013858
19	9	0	3.278680	-2.198065	-0.185349
20	9	0	3.876122	-0.525612	1.065825
21	8	0	1.921811	1.949383	0.835003
22	1	0	2.467680	2.409655	0.171988
23	1	0	2.343327	2.112927	1.684183
24	8	0	3.598170	2.803782	-1.227423
25	1	0	3.439088	3.414697	-1.953600
26	1	0	3.787218	1.948049	-1.629017

2 water Transition state

Center Number	Atomic Number	Atomic Type	Coordinates (Angstroms) X	Y	Z
1	6	0	-2.666456	-1.059514	0.441571
2	6	0	-3.324254	0.055500	-0.068253
3	6	0	-2.657784	1.046917	-0.780232
4	6	0	-1.290109	0.917059	-0.980661
5	6	0	-0.627919	-0.195295	-0.471895
6	6	0	-1.299667	-1.189591	0.234469
7	1	0	-3.225761	-1.806917	0.985848
8	1	0	-3.207818	1.895795	-1.160205
9	1	0	-0.728672	1.663921	-1.525824
10	1	0	-0.753908	-2.037892	0.620149

11	7	0	-4.776800	0.189926	0.151093
12	8	0	-5.341995	-0.696328	0.779653
13	8	0	-5.332182	1.180575	-0.308596
14	8	0	0.725026	-0.312971	-0.765190
15	6	0	1.628206	-0.289168	0.270431
16	8	0	1.329458	-0.776765	1.440461
17	6	0	2.985343	-0.712134	-0.356753
18	1	0	1.536644	0.486508	1.714998
19	9	0	3.308984	0.024924	-1.436574
20	9	0	2.947457	-1.996841	-0.717084
21	9	0	3.984889	-0.560550	0.541781
22	1	0	2.677301	1.629389	0.763936
23	8	0	1.774152	1.218505	0.803887
24	8	0	4.233976	2.318293	0.741516
25	1	0	4.449264	3.075174	0.186640
26	1	0	4.926949	1.663624	0.597806

2 water Intermediate 2

Center Number	Atomic Number	Atomic Type	Coordinates (Angstroms) X	Y	Z
1	6	0	-2.493812	-0.912138	-0.930392
2	6	0	-3.086995	0.080028	-0.154740
3	6	0	-2.332857	1.077395	0.453313
4	6	0	-0.953791	1.091231	0.292059
5	6	0	-0.348369	0.091125	-0.472793
6	6	0	-1.116981	-0.904545	-1.084950
7	1	0	-3.109864	-1.668162	-1.396276
8	1	0	-2.830516	1.836141	1.040578
9	1	0	-0.360325	1.865854	0.749884
10	1	0	-0.617055	-1.652794	-1.686732
11	7	0	-4.546672	0.072637	0.024310
12	8	0	-5.180779	-0.829633	-0.511364
13	8	0	-5.043062	0.966622	0.699448
14	8	0	0.998002	-0.002575	-0.712504
15	6	0	1.940084	0.606611	0.136761
16	8	0	1.726951	0.384842	1.472835
17	6	0	3.292292	-0.033494	-0.319630
18	1	0	1.527001	-0.566883	1.635083
19	9	0	3.508283	0.104413	-1.629284
20	9	0	3.322086	-1.356523	-0.028769
21	9	0	4.308895	0.549146	0.339776
22	1	0	2.407911	2.394099	0.643300
23	8	0	1.966460	1.970386	-0.104215
24	8	0	0.980796	-2.216515	1.725624
25	1	0	0.062265	-2.360673	1.470970
26	1	0	1.509549	-2.791648	1.161172

3 water Intermediate 1

Center Number	Atomic Number	Atomic Type	Coordinates (Angstroms) X	Y	Z
1	6	0	2.963572	-1.491701	0.014753
2	6	0	3.367729	-0.160922	0.012361
3	6	0	2.465212	0.889060	0.134984
4	6	0	1.107936	0.611415	0.260504
5	6	0	0.712938	-0.718707	0.254174
6	6	0	1.610056	-1.773434	0.139396
7	1	0	3.698767	-2.277859	-0.078590
8	1	0	2.820851	1.909549	0.136572
9	1	0	0.400427	1.426899	0.361988
10	1	0	1.246513	-2.792509	0.151506
11	7	0	4.807793	0.143066	-0.122102
12	8	0	5.580255	-0.803011	-0.214289
13	8	0	5.140451	1.320290	-0.133984
14	8	0	-0.632488	-1.089270	0.442207
15	6	0	-1.586397	-0.600916	-0.343424
16	8	0	-1.468468	0.185678	-1.240234
17	6	0	-2.923951	-1.317598	-0.024934
18	1	0	-3.562038	1.446780	1.963040
19	9	0	-3.126551	-1.499149	1.281324
20	9	0	-2.898552	-2.531993	-0.616883
21	9	0	-3.953497	-0.634408	-0.534203
22	1	0	-3.257937	1.951033	0.522893
23	8	0	-2.862902	1.532666	1.308371
24	8	0	-2.950789	2.582564	-1.255325
25	1	0	-2.524534	1.776513	-1.589471
26	1	0	-3.541889	2.892003	-1.948053
27	8	0	-0.671587	3.244046	0.644491
28	1	0	-1.188293	3.457582	-0.142769
29	1	0	-1.317380	2.791608	1.208666

3 water Transition state

Center Number	Atomic Number	Atomic Type	Coordinates (Angstroms) X	Y	Z
1	6	0	-2.407821	-0.770998	-0.994252
2	6	0	-2.960582	0.156950	-0.118900
3	6	0	-2.196359	1.127286	0.519626
4	6	0	-0.824906	1.164304	0.293114
5	6	0	-0.279068	0.211793	-0.554474
6	6	0	-1.038130	-0.738643	-1.222891

7	1	0	-3.044390	-1.494585	-1.482651
8	1	0	-2.674708	1.844138	1.171663
9	1	0	-0.188850	1.930740	0.725133
10	1	0	-0.569736	-1.436796	-1.902723
11	7	0	-4.418855	0.118015	0.127215
12	8	0	-5.069404	-0.734445	-0.463202
13	8	0	-4.883292	0.937859	0.907108
14	8	0	1.103602	0.301765	-0.829296
15	6	0	2.045791	-0.355794	-0.180230
16	8	0	2.435224	0.441076	1.420575
17	6	0	1.733346	-1.803439	0.301125
18	1	0	3.215532	1.779230	-1.464326
19	9	0	0.685232	-1.857881	1.136560
20	9	0	2.791992	-2.319408	0.932760
21	9	0	1.465441	-2.570225	-0.769567
22	1	0	3.853073	3.140745	-1.812860
23	8	0	3.042455	2.731587	-1.496204
24	8	0	3.263384	-0.095259	-0.543661
25	1	0	3.389893	0.285563	0.586248
26	1	0	2.424617	-0.091310	2.228760
27	8	0	1.561525	3.205297	0.958090
28	1	0	2.012749	3.256081	0.099433
29	1	0	1.968246	2.435925	1.377049

3 water Intermediate 2

Center Number	Atomic Number	Atomic Type	Coordinates (Angstroms) X	Y	Z
1	6	0	2.415168	-1.406730	0.505742
2	6	0	3.129851	-0.284603	0.095561
3	6	0	2.509366	0.926094	-0.187759
4	6	0	1.126937	1.022477	-0.062043
5	6	0	0.409951	-0.098092	0.346182
6	6	0	1.037211	-1.308552	0.631722
7	1	0	2.939203	-2.327880	0.716709
8	1	0	3.102565	1.774904	-0.496644
9	1	0	0.624809	1.961058	-0.266172
10	1	0	0.440680	-2.156164	0.941417
11	7	0	4.596587	-0.385861	-0.041497
12	8	0	5.118752	-1.462259	0.221495
13	8	0	5.203584	0.611257	-0.411127
14	8	0	-0.967313	-0.003754	0.529173
15	6	0	-1.815878	-0.337342	-0.591706
16	8	0	-2.229158	0.798684	-1.245710
17	6	0	-3.007243	-1.101276	0.059217
18	1	0	-1.792588	1.678803	1.378224
19	9	0	-2.601557	-2.233222	0.650703
20	9	0	-3.931515	-1.408819	-0.855650

21	9	0	-3.599988	-0.330714	1.001564
22	1	0	-3.070655	2.522731	1.615026
23	8	0	-2.303764	2.432253	1.039496
24	8	0	-1.222346	-1.187739	-1.481722
25	1	0	-2.407840	1.502606	-0.590949
26	1	0	-0.929252	-0.662232	-2.237524
27	8	0	-0.247937	3.967885	-0.182539
28	1	0	-0.468809	4.704187	-0.759471
29	1	0	-1.063412	3.735340	0.286291

4 water Intermediate 1

Center Number	Atomic Number	Atomic Type	Coordinates (Angstroms) X	Y	Z
1	6	0	-2.811032	-0.101867	-1.226489
2	6	0	-3.139559	-0.024376	0.122841
3	6	0	-2.186054	0.201529	1.109422
4	6	0	-0.855126	0.351545	0.741401
5	6	0	-0.535660	0.251756	-0.604091
6	6	0	-1.481262	0.043958	-1.596136
7	1	0	-3.586676	-0.269050	-1.959629
8	1	0	-2.490982	0.266680	2.143947
9	1	0	-0.075547	0.547585	1.466357
10	1	0	-1.177564	0.005504	-2.633456
11	7	0	-4.553836	-0.179863	0.519560
12	8	0	-5.374557	-0.374512	-0.368305
13	8	0	-4.820576	-0.105486	1.712043
14	8	0	0.789857	0.478246	-1.024904
15	6	0	1.822319	-0.302971	-0.759909
16	8	0	4.549908	-0.328272	1.425492
17	6	0	1.557922	-1.768624	-0.286806
18	1	0	3.012303	2.032836	-0.749258
19	9	0	1.061948	-1.836833	0.950389
20	9	0	2.707658	-2.451718	-0.313511
21	9	0	0.695792	-2.367424	-1.129337
22	1	0	3.522396	3.459073	-0.420855
23	8	0	2.791482	2.850939	-0.273408
24	8	0	2.950362	0.060959	-0.979554
25	1	0	4.360999	-0.485236	0.490272
26	1	0	5.002804	-1.114745	1.744027
27	8	0	2.045200	0.990800	1.859183
28	1	0	2.293872	1.819355	1.424265
29	1	0	2.889399	0.536244	2.010631
30	8	0	-0.041286	3.321507	-0.722367
31	1	0	0.919212	3.267637	-0.595397
32	1	0	-0.334365	4.037789	-0.152541

4 water Transition state

Center Number	Atomic Number	Atomic Type	Coordinates (Angstroms) X	Y	Z
1	6	0	2.133359	-1.348969	0.421495
2	6	0	2.994769	-0.490257	-0.254056
3	6	0	2.517871	0.513172	-1.091972
4	6	0	1.148915	0.667572	-1.237775
5	6	0	0.276773	-0.179810	-0.554419
6	6	0	0.761659	-1.199881	0.267046
7	1	0	2.541059	-2.126436	1.052132
8	1	0	3.215060	1.161787	-1.602728
9	1	0	0.743190	1.452411	-1.860694
10	1	0	0.083329	-1.859949	0.785047
11	7	0	4.447828	-0.649568	-0.081255
12	8	0	4.837338	-1.513146	0.696077
13	8	0	5.181690	0.093785	-0.721944
14	8	0	-1.062372	0.038765	-0.786297
15	6	0	-2.023176	-0.196786	0.218539
16	8	0	-2.978590	0.938829	0.214161
17	6	0	-2.835204	-1.462323	-0.170292
18	1	0	-0.208789	0.867643	2.034795
19	9	0	-3.329836	-1.362885	-1.414580
20	9	0	-3.852207	-1.656890	0.674088
21	9	0	-2.038594	-2.549636	-0.126538
22	1	0	1.311802	1.087074	2.221051
23	8	0	0.472215	1.556064	2.181232
24	8	0	-1.656855	-0.111021	1.495189
25	1	0	-2.539458	0.867587	1.323882
26	1	0	-2.637996	1.750644	-0.337527
27	8	0	0.115751	3.517673	0.350776
28	1	0	0.312175	2.819560	1.022224
29	1	0	0.222881	4.361108	0.800825
30	8	0	-2.131775	3.006364	-0.957484
31	1	0	-1.986166	2.989293	-1.908281
32	1	0	-1.275175	3.266153	-0.523740

4 water Intermediate 2

Center Number	Atomic Number	Atomic Type	Coordinates (Angstroms) X	Y	Z
1	6	0	-2.731708	-0.280083	-1.065867
2	6	0	-3.228721	0.375378	0.056109
3	6	0	-2.390161	0.947959	1.006352
4	6	0	-1.017407	0.854470	0.826311
5	6	0	-0.511464	0.201837	-0.296867

6	6	0	-1.358658	-0.361163	-1.251744
7	1	0	-3.419922	-0.709468	-1.779992
8	1	0	-2.815210	1.444076	1.867190
9	1	0	-0.325256	1.270697	1.546763
10	1	0	-0.943753	-0.865214	-2.111505
11	7	0	-4.688070	0.468951	0.241931
12	8	0	-5.404065	-0.050797	-0.605231
13	8	0	-5.098019	1.062663	1.232105
14	8	0	0.866349	0.225716	-0.442763
15	6	0	1.657804	-0.967234	-0.660445
16	8	0	2.950783	-0.573409	-0.698302
17	6	0	1.388342	-1.973400	0.506548
18	1	0	1.460365	1.908118	-1.039233
19	9	0	1.572344	-1.372010	1.696711
20	9	0	2.236654	-3.005776	0.428312
21	9	0	0.135491	-2.460984	0.480833
22	1	0	0.848178	3.302948	-1.297210
23	8	0	1.681845	2.822972	-1.287857
24	8	0	1.326599	-1.607955	-1.835296
25	1	0	2.000928	-1.361737	-2.482107
26	1	0	3.209414	-0.018731	0.093462
27	8	0	3.854216	3.465965	0.240531
28	1	0	3.078678	3.378683	-0.354965
29	1	0	4.583962	3.767331	-0.308352
30	8	0	3.661631	0.989918	1.269120
31	1	0	4.212087	0.767305	2.024002
32	1	0	3.863738	1.915084	0.994885

5 water Intermediate 1

Center Number	Atomic Number	Atomic Type	Coordinates (Angstroms) X	Y	Z
1	6	0	2.035314	-1.981074	0.630241
2	6	0	2.728094	-1.099814	-0.193423
3	6	0	2.112239	-0.389951	-1.216662
4	6	0	0.742874	-0.524296	-1.390451
5	6	0	0.039706	-1.340416	-0.518561
6	6	0	0.660742	-2.096181	0.469590
7	1	0	2.563138	-2.531396	1.396636
8	1	0	2.683798	0.276261	-1.845504
9	1	0	0.223296	0.041057	-2.149172
10	1	0	0.075550	-2.735297	1.116946
11	7	0	4.149472	-0.850022	0.079326
12	8	0	4.796282	-1.725886	0.630718
13	8	0	4.593971	0.252626	-0.243599
14	8	0	-1.350554	-1.370900	-0.666176
15	6	0	-2.070287	-0.764955	0.285018
16	8	0	-1.741396	1.594936	-0.823176

17	6	0	-3.571636	-0.778837	-0.087875
18	1	0	-0.170345	0.625372	1.768189
19	9	0	-3.785680	-0.713843	-1.399624
20	9	0	-4.209519	0.218938	0.516541
21	9	0	-4.092690	-1.948048	0.363058
22	1	0	0.684111	1.087200	2.972330
23	8	0	0.502671	1.276154	2.045920
24	8	0	-1.676738	-0.374145	1.349202
25	1	0	-1.811611	2.255565	-0.112843
26	1	0	-1.000953	1.920715	-1.362520
27	8	0	2.321021	2.429268	0.191646
28	1	0	1.797957	1.959023	0.865391
29	1	0	3.150766	1.941175	0.103330
30	8	0	0.701199	2.510530	-2.041505
31	1	0	0.764645	3.349903	-2.505825
32	1	0	1.317407	2.568084	-1.278632
33	8	0	-1.287225	3.398309	1.355413
34	1	0	-0.573409	2.844323	1.713049
35	1	0	-0.895775	4.260762	1.188760

5 water Transition state

Center Number	Atomic Number	Atomic Type	Coordinates (Angstroms) X	Y	Z
1	6	0	2.518934	-1.223886	0.476398
2	6	0	3.217680	-0.245121	-0.223098
3	6	0	2.578125	0.623142	-1.102155
4	6	0	1.206495	0.516262	-1.267201
5	6	0	0.496005	-0.453383	-0.561231
6	6	0	1.146420	-1.336695	0.302287
7	1	0	3.052531	-1.889257	1.140373
8	1	0	3.151556	1.372762	-1.628226
9	1	0	0.674463	1.193376	-1.920400
10	1	0	0.592642	-2.093124	0.836564
11	7	0	4.672439	-0.127167	-0.030535
12	8	0	5.200803	-0.873661	0.785309
13	8	0	5.268150	0.712702	-0.694586
14	8	0	-0.858706	-0.502990	-0.814025
15	6	0	-1.769413	-0.840575	0.207170
16	8	0	-2.909265	0.122692	0.144552
17	6	0	-2.361022	-2.240194	-0.113105
18	1	0	-0.166518	0.639392	1.967221
19	9	0	-2.870442	-2.283797	-1.355356
20	9	0	-3.328357	-2.561584	0.750633
21	9	0	-1.393842	-3.175433	-0.028905
22	1	0	1.255926	1.195782	2.226169
23	8	0	0.345187	1.465071	2.072410
24	8	0	-1.432427	-0.622520	1.471606

25	1	0	-2.459568	0.196656	1.246121
26	1	0	-2.719104	0.927913	-0.470321
27	8	0	-0.357075	3.158293	0.037075
28	1	0	-0.026041	2.597207	0.774294
29	1	0	-0.837308	3.898075	0.442290
30	8	0	-2.716721	4.599091	0.434605
31	1	0	-3.256037	5.393981	0.468610
32	1	0	-3.110384	4.018638	-0.231437
33	8	0	-2.404240	2.214320	-1.219114
34	1	0	-1.529928	2.539428	-0.824243
35	1	0	-2.280514	2.105360	-2.167666

5 water Intermediate 2

Center Number	Atomic Number	Atomic Type	Coordinates (Angstroms) X	Y	Z
1	6	0	2.301350	-1.396378	0.510645
2	6	0	3.189926	-0.539627	-0.132836
3	6	0	2.757723	0.387213	-1.076633
4	6	0	1.404300	0.466871	-1.361923
5	6	0	0.501031	-0.377112	-0.713323
6	6	0	0.947186	-1.322329	0.214008
7	1	0	2.676871	-2.114663	1.225854
8	1	0	3.473923	1.037896	-1.557539
9	1	0	1.026218	1.191869	-2.069113
10	1	0	0.247912	-1.981715	0.702967
11	7	0	4.623863	-0.620455	0.189380
12	8	0	4.966272	-1.400580	1.070855
13	8	0	5.390219	0.099785	-0.439237
14	8	0	-0.809805	-0.242399	-1.103388
15	6	0	-1.903660	-0.211205	-0.204649
16	8	0	-2.792760	0.737108	-0.621786
17	6	0	-2.653200	-1.578526	-0.303660
18	1	0	0.054524	0.986762	1.676586
19	9	0	-3.051045	-1.835457	-1.552226
20	9	0	-3.742813	-1.573420	0.493545
21	9	0	-1.867191	-2.597092	0.097929
22	1	0	1.566487	1.066357	1.954999
23	8	0	0.769642	1.605227	1.909495
24	8	0	-1.449818	-0.010910	1.098711
25	1	0	-2.208561	0.355986	1.618382
26	1	0	-2.306175	1.563604	-0.912701
27	8	0	0.595064	3.575895	0.042176
28	1	0	0.738832	2.893270	0.737682
29	1	0	0.647084	4.425679	0.489912
30	8	0	-1.628786	2.971864	-1.344291
31	1	0	-1.453220	3.107737	-2.279764
32	1	0	-0.814107	3.245191	-0.855539

33	8	0	-3.665533	1.171298	2.056195
34	1	0	-4.381201	0.719560	2.515126
35	1	0	-3.950078	1.267612	1.134635

11 water Intermediate 1

Center Number	Atomic Number	Atomic Type	Coordinates (Angstroms) X	Y	Z
1	6	0	-0.067456	-3.113331	-0.534657
2	6	0	-0.890163	-2.695529	0.507165
3	6	0	-0.414649	-1.952562	1.582920
4	6	0	0.918932	-1.580298	1.592435
5	6	0	1.727389	-1.947937	0.524570
6	6	0	1.266665	-2.735219	-0.525696
7	1	0	-0.480845	-3.688251	-1.350026
8	1	0	-1.088361	-1.623984	2.359707
9	1	0	1.323284	-0.957680	2.377333
10	1	0	1.926685	-3.015444	-1.333553
11	7	0	-2.321551	-2.946698	0.412687
12	8	0	-2.733219	-3.612251	-0.542538
13	8	0	-3.060626	-2.465628	1.264273
14	8	0	3.041506	-1.485811	0.570305
15	6	0	3.505279	-0.741100	-0.445455
16	8	0	1.772767	1.250303	0.537462
17	6	0	4.871783	-0.125046	-0.053089
18	1	0	0.983884	1.045450	-0.959005
19	9	0	4.966879	0.151662	1.248672
20	9	0	5.080134	0.996585	-0.747254
21	9	0	5.849655	-1.004198	-0.368112
22	1	0	0.973374	0.561313	-2.429612
23	8	0	0.427111	1.021340	-1.783302
24	8	0	3.025276	-0.595911	-1.528784
25	1	0	2.126534	2.164972	0.502520
26	1	0	0.963309	1.294705	1.094908
27	8	0	-1.805156	-0.082480	-0.550823
28	1	0	-1.182033	0.296617	-1.200114
29	1	0	-2.716905	0.112284	-0.811891
30	8	0	-0.748376	1.274160	1.657528
31	1	0	-1.079444	2.181859	1.532580
32	1	0	-1.201977	0.760939	0.958860
33	8	0	2.392044	3.898881	0.131583
34	1	0	1.638182	4.016962	-0.495159
35	1	0	3.175240	4.244737	-0.305014
36	8	0	-4.165231	4.221431	0.142826
37	1	0	-4.302391	4.659556	-0.702171
38	1	0	-4.787501	3.457817	0.154400
39	8	0	-5.866079	2.115424	0.068038
40	1	0	-5.467671	1.220998	-0.013040

41	1	0	-6.605480	2.032576	0.676442
42	8	0	-4.614718	-0.296615	-0.178696
43	1	0	-4.986048	-1.050830	-0.686043
44	1	0	-4.261477	-0.707810	0.621890
45	8	0	-5.313936	-2.680959	-1.339472
46	1	0	-5.464289	-2.888298	-2.266399
47	1	0	-4.532646	-3.194095	-1.067713
48	8	0	-1.605282	3.882738	0.875083
49	1	0	-1.386121	4.612227	1.463796
50	1	0	-2.558942	3.997385	0.636380
51	8	0	0.136610	3.848233	-1.369292
52	1	0	0.140190	2.935763	-1.707354
53	1	0	-0.535893	3.863967	-0.662982

11 water Transition state

Center Number	Atomic Number	Atomic Type	Coordinates (Angstroms) X	Y	Z
1	6	0	0.391240	-2.560033	1.060638
2	6	0	1.213082	-2.675382	-0.058303
3	6	0	0.755732	-2.395596	-1.343518
4	6	0	-0.542846	-1.947848	-1.503331
5	6	0	-1.354573	-1.775972	-0.382430
6	6	0	-0.910357	-2.116562	0.897086
7	1	0	0.778998	-2.808150	2.038115
8	1	0	1.422032	-2.500179	-2.187098
9	1	0	-0.932152	-1.685746	-2.477421
10	1	0	-1.576596	-2.019333	1.740988
11	7	0	2.607235	-3.037599	0.131687
12	8	0	3.007685	-3.276356	1.267816
13	8	0	3.344369	-3.063046	-0.861752
14	8	0	-2.635580	-1.345693	-0.625109
15	6	0	-3.212419	-0.279879	0.072126
16	8	0	-3.028530	1.016092	-0.624987
17	6	0	-4.733913	-0.588692	0.075242
18	1	0	-1.123670	0.562792	1.945657
19	9	0	-5.205592	-0.808475	-1.161901
20	9	0	-5.419395	0.430310	0.609228
21	9	0	-4.970478	-1.687325	0.815462
22	1	0	-0.151578	0.943682	3.104955
23	8	0	-0.306468	1.058359	2.162359
24	8	0	-2.726346	0.103447	1.263984
25	1	0	-2.713912	1.179761	0.535809
26	1	0	-2.140158	1.044614	-1.213942
27	8	0	1.119811	0.540561	-0.217610
28	1	0	0.904598	0.774225	0.699615
29	1	0	2.021250	0.852113	-0.423112
30	8	0	-0.879821	1.240073	-1.862668

31	1	0	-0.714591	2.207074	-1.914374
32	1	0	-0.126783	0.912657	-1.305184
33	8	0	-0.215903	3.861253	-1.425923
34	1	0	-0.363818	3.848433	-0.435462
35	1	0	-0.674260	4.636867	-1.764289
36	8	0	2.622872	4.033316	-0.956016
37	1	0	1.715925	3.957757	-1.301411
38	1	0	3.029475	3.155474	-1.065003
39	8	0	3.610581	1.372118	-1.156513
40	1	0	4.404759	1.064477	-0.651250
41	1	0	3.730822	1.044800	-2.053813
42	8	0	5.709156	0.418289	0.198611
43	1	0	5.785243	-0.559118	0.297106
44	1	0	6.602601	0.771357	0.178684
45	8	0	5.784617	-2.289047	0.419795
46	1	0	5.506549	-2.623323	1.280421
47	1	0	5.133716	-2.678673	-0.189056
48	8	0	2.133519	4.660524	1.625576
49	1	0	2.454093	5.534773	1.862900
50	1	0	2.441826	4.484798	0.703494
51	8	0	-0.409695	3.764796	1.206185
52	1	0	-0.421925	2.871055	1.589389
53	1	0	0.438286	4.162722	1.502319

11 water Intermediate 2

Center Number	Atomic Number	Atomic Type	Coordinates (Angstroms) X	Y	Z
1	6	0	0.453318	-2.836180	1.263289
2	6	0	1.211592	-2.709151	0.098517
3	6	0	0.645716	-2.307353	-1.108720
4	6	0	-0.702328	-1.994421	-1.151507
5	6	0	-1.458431	-2.050028	0.023261
6	6	0	-0.888987	-2.503913	1.219488
7	1	0	0.924148	-3.170285	2.177064
8	1	0	1.263850	-2.228324	-1.990226
9	1	0	-1.167915	-1.686566	-2.074946
10	1	0	-1.516302	-2.568952	2.099225
11	7	0	2.640564	-2.955807	0.161945
12	8	0	3.145132	-3.234266	1.247997
13	8	0	3.299710	-2.852131	-0.879396
14	8	0	-2.780525	-1.713509	0.056352
15	6	0	-3.137306	-0.374726	-0.309603
16	8	0	-2.927281	-0.077221	-1.614057
17	6	0	-4.672428	-0.336120	-0.046164
18	1	0	-0.887663	0.462859	1.461685
19	9	0	-5.344275	-1.144797	-0.874836
20	9	0	-5.120562	0.923527	-0.234174

21	9	0	-4.970077	-0.686992	1.215151
22	1	0	0.151452	0.092380	2.550674
23	8	0	-0.018436	0.714290	1.836670
24	8	0	-2.487411	0.478220	0.580912
25	1	0	-2.555015	1.424932	0.284776
26	1	0	-2.087246	0.459825	-1.751009
27	8	0	1.376918	0.686543	-0.628261
28	1	0	1.109879	0.717044	0.305927
29	1	0	2.267046	1.072016	-0.706262
30	8	0	-0.851249	1.452624	-2.014559
31	1	0	-1.161003	2.251401	-1.557373
32	1	0	-0.004091	1.204540	-1.580574
33	8	0	-2.216300	3.041741	-0.095764
34	1	0	-1.577796	3.355253	0.612459
35	1	0	-2.945311	3.668174	-0.134338
36	8	0	3.442305	4.373383	-0.883600
37	1	0	3.206362	4.747598	-1.737363
38	1	0	3.652128	3.430707	-1.045585
39	8	0	3.949841	1.669348	-1.269429
40	1	0	4.702017	1.277304	-0.754547
41	1	0	4.052731	1.342404	-2.169304
42	8	0	5.928839	0.539076	0.112355
43	1	0	5.933555	-0.445416	0.176918
44	1	0	6.846559	0.821704	0.072823
45	8	0	5.837320	-2.163861	0.227710
46	1	0	5.629982	-2.538631	1.091565
47	1	0	5.114246	-2.494168	-0.334223
48	8	0	1.764173	4.981848	1.184291
49	1	0	2.284071	5.469905	1.828016
50	1	0	2.371094	4.754148	0.446773
51	8	0	-0.469318	3.531405	1.779229
52	1	0	-0.166168	2.636883	2.004709
53	1	0	0.333038	4.059712	1.579280

Table S4. Cartesian coordinates of each of the intermediates and transition states studied for S-ethyl trifluorothioacetate. Naming follows the convention in Figures 4 and 5 in the manuscript.

<u>S-ethyl trifluorothioacetate</u>

1 water Intermediate 1

Center Number	Atomic Number	Atomic Type	Coordinates (Angstroms) X	Y	Z
1	6	0	-1.949366	-0.052744	0.017091
2	9	0	-2.204715	-1.230343	0.613444
3	9	0	-2.552124	-0.054899	-1.182960
4	9	0	-2.493578	0.918896	0.761836
5	6	0	-0.427129	0.205156	-0.164323
6	8	0	-0.070313	1.284528	-0.574203
7	16	0	0.596241	-1.159968	0.235440
8	6	0	2.239109	-0.454421	-0.206483
9	1	0	2.441749	0.377645	0.465601
10	1	0	2.158074	-0.063734	-1.220733
11	6	0	3.298486	-1.544734	-0.100360
12	1	0	4.270223	-1.118235	-0.359420
13	1	0	3.368532	-1.941948	0.914813
14	1	0	3.099561	-2.373894	-0.783055
15	8	0	2.293965	2.714470	0.293111
16	1	0	1.457316	2.438465	-0.107931
17	1	0	2.162622	3.626790	0.565994

1 water Transition state

Center Number	Atomic Number	Atomic Type	Coordinates (Angstroms) X	Y	Z
1	6	0	1.595481	-0.408694	0.075590
2	9	0	1.734442	-1.046231	-1.101207
3	9	0	1.658608	-1.319602	1.057020
4	9	0	2.645037	0.420793	0.219399
5	6	0	0.252681	0.360534	0.160505
6	8	0	0.105106	1.194540	1.150659
7	16	0	-1.101929	-0.610898	-0.431502
8	6	0	-2.499836	0.363546	0.261782
9	1	0	-2.516923	1.325388	-0.250577
10	1	0	-2.288930	0.530815	1.316025
11	6	0	-3.797291	-0.408099	0.048058
12	1	0	-4.631125	0.171100	0.452018
13	1	0	-3.994582	-0.584361	-1.011841

14	1	0	-3.777645	-1.372921	0.559718
15	8	0	0.560074	1.789864	-0.909933
16	1	0	0.412757	2.017953	0.324751
17	1	0	1.456848	1.872799	-1.264393

1 water Intermediate 2

Center Number	Atomic Number	Atomic Type	Coordinates (Angstroms) X	Y	Z
1	6	0	1.628783	-0.308078	0.000034
2	9	0	1.795542	-1.069291	-1.084250
3	9	0	1.795547	-1.069152	1.084359
4	9	0	2.634282	0.623726	-0.000032
5	6	0	0.276969	0.458233	-0.000027
6	8	0	0.199650	1.232270	1.166943
7	16	0	-1.095313	-0.745955	-0.000010
8	6	0	-2.495356	0.449061	0.000007
9	1	0	-2.403501	1.072823	-0.888081
10	1	0	-2.403494	1.072834	0.888088
11	6	0	-3.805360	-0.331670	0.000011
12	1	0	-4.646207	0.366547	0.000076
13	1	0	-3.893727	-0.965082	-0.885753
14	1	0	-3.893681	-0.965167	0.885722
15	8	0	0.199683	1.232208	-1.166998
16	1	0	0.956077	1.832506	1.211955
17	1	0	0.956311	1.832175	-1.212248

2 water Intermediate 1

Center Number	Atomic Number	Atomic Type	Coordinates (Angstroms) X	Y	Z
1	6	0	-2.118121	0.025221	0.126022
2	9	0	-2.473100	-0.952312	0.976317
3	9	0	-2.915540	-0.049950	-0.951901
4	9	0	-2.338182	1.203854	0.729574
5	6	0	-0.630630	-0.070109	-0.312485
6	8	0	-0.179312	0.813298	-1.006640
7	16	0	0.213286	-1.488770	0.252780
8	6	0	1.868467	-1.174559	-0.491291
9	1	0	2.290276	-0.304929	0.013660
10	1	0	1.705607	-0.940209	-1.543184
11	6	0	2.742372	-2.409555	-0.307266
12	1	0	3.725882	-2.212493	-0.740455
13	1	0	2.887413	-2.644263	0.749620

14	1	0	2.323453	-3.287740	-0.804093
15	8	0	3.003258	1.449504	1.084083
16	1	0	2.531703	2.151992	0.597210
17	1	0	3.881836	1.797589	1.258152
18	8	0	1.418783	3.112660	-0.505715
19	1	0	0.836626	2.416803	-0.849143
20	1	0	0.831652	3.799546	-0.175860

2 water Transition state

Center Number	Atomic Number	Atomic Type	Coordinates (Angstroms) X	Y	Z
1	6	0	1.600844	-0.376283	-0.203082
2	9	0	1.860878	0.277569	-1.353557
3	9	0	1.534310	-1.689922	-0.474430
4	9	0	2.633019	-0.178969	0.627182
5	6	0	0.270291	0.100695	0.430906
6	8	0	-0.000962	-0.350679	1.628547
7	16	0	-1.086616	0.022010	-0.867178
8	6	0	-2.364121	-0.850164	0.127622
9	1	0	-3.302466	-0.600641	-0.372963
10	1	0	-2.367304	-0.402137	1.120404
11	6	0	-2.142673	-2.354410	0.198923
12	1	0	-2.958133	-2.821000	0.760446
13	1	0	-2.112777	-2.797868	-0.798425
14	1	0	-1.206359	-2.577968	0.710443
15	8	0	0.568383	1.570045	0.879658
16	1	0	0.248435	0.944263	1.829332
17	1	0	-0.092694	2.233699	0.535980
18	8	0	-1.268548	3.134528	-0.241983
19	1	0	-1.976320	3.656730	0.148861
20	1	0	-1.677413	2.384480	-0.707968

2 water Intermediate 2

Center Number	Atomic Number	Atomic Type	Coordinates (Angstroms) X	Y	Z
1	6	0	-1.281848	-0.980925	-0.078028
2	9	0	-1.269563	-1.809374	0.990985
3	9	0	-1.152057	-1.724818	-1.179871
4	9	0	-2.500092	-0.397419	-0.118644
5	6	0	-0.173288	0.107810	0.045086
6	8	0	-0.220731	0.951812	-1.031202
7	16	0	1.459836	-0.751794	0.134088

8	6	0	2.540824	0.708754	-0.156582
9	1	0	2.300084	1.467546	0.588304
10	1	0	2.302867	1.100244	-1.143660
11	6	0	3.999554	0.275530	-0.056618
12	1	0	4.648674	1.134956	-0.242649
13	1	0	4.236443	-0.118213	0.934592
14	1	0	4.238805	-0.492386	-0.796000
15	8	0	-0.439482	0.851384	1.229522
16	1	0	-0.901762	1.644232	-0.868188
17	1	0	-0.110550	0.348733	1.984658
18	8	0	-2.028196	2.776036	-0.068661
19	1	0	-1.809387	2.400260	0.796448
20	1	0	-2.971316	2.626955	-0.191574

3 water Intermediate 1

Center Number	Atomic Number	Atomic Type	Coordinates (Angstroms) X	Y	Z
1	6	0	1.698378	-0.824200	0.084668
2	9	0	2.112707	-0.117332	-0.981892
3	9	0	1.996594	-2.120961	-0.144436
4	9	0	2.384126	-0.425019	1.155202
5	6	0	0.166190	-0.697454	0.314437
6	8	0	-0.297675	-0.861606	1.407166
7	16	0	-0.756003	-0.437910	-1.185164
8	6	0	-2.464209	-0.434154	-0.499189
9	1	0	-3.055457	0.012543	-1.301223
10	1	0	-2.455868	0.256305	0.345476
11	6	0	-2.974484	-1.821533	-0.133959
12	1	0	-4.004058	-1.741105	0.226594
13	1	0	-2.966266	-2.492582	-0.995449
14	1	0	-2.374167	-2.263190	0.661357
15	8	0	0.752085	2.165094	0.836524
16	1	0	1.548085	2.502155	1.257275
17	1	0	0.689103	2.587214	-0.041944
18	8	0	0.203959	2.797847	-1.783766
19	1	0	-0.513814	3.390656	-2.028517
20	1	0	-0.133673	1.899770	-1.918428
21	8	0	-1.795498	1.722331	2.004707
22	1	0	-1.621698	0.918680	2.505651
23	1	0	-0.915205	1.980646	1.679177

3 water Transition state

Center Number	Atomic Number	Atomic Type	Coordinates (Angstroms) X	Y	Z

1	6	0	-1.837096	-0.420594	0.110005
2	9	0	-2.314064	-0.795468	-1.090198
3	9	0	-2.351352	0.783688	0.409482
4	9	0	-2.286284	-1.296378	1.023488
5	6	0	-0.286156	-0.357023	0.116754
6	8	0	0.237140	-0.189207	1.340039
7	16	0	0.314516	0.699411	-1.281551
8	6	0	1.085455	2.094125	-0.360801
9	1	0	1.577009	2.649258	-1.164234
10	1	0	1.865390	1.695677	0.284013
11	6	0	0.102880	2.974385	0.397228
12	1	0	0.639640	3.817500	0.843524
13	1	0	-0.668751	3.369186	-0.266291
14	1	0	-0.381555	2.417344	1.198782
15	8	0	0.135122	-1.811729	-0.051312
16	1	0	0.410035	-1.475916	1.063409
17	1	0	1.023727	-1.853901	-0.546330
18	8	0	2.572294	-1.828519	-0.947412
19	1	0	2.953177	-1.311156	-0.204157
20	1	0	2.688054	-1.279468	-1.731069
21	8	0	2.995375	-0.259649	1.258532
22	1	0	2.053083	-0.092201	1.475490
23	1	0	3.463288	-0.385992	2.088841

3 water Intermediate 2

Center Number	Atomic Number	Atomic Type	Coordinates (Angstroms) X	Y	Z
1	6	0	-1.756936	-0.598356	0.124838
2	9	0	-2.318767	-1.053811	-0.995484
3	9	0	-2.303557	0.594084	0.415428
4	9	0	-2.097228	-1.440027	1.137325
5	6	0	-0.207433	-0.525305	0.017653
6	8	0	0.294155	-0.107996	1.286420
7	16	0	0.330056	0.699134	-1.268200
8	6	0	0.742713	2.242413	-0.353471
9	1	0	1.236880	2.823985	-1.136992
10	1	0	1.499179	2.009117	0.393436
11	6	0	-0.425749	3.017496	0.237213
12	1	0	-0.065295	3.976354	0.623371
13	1	0	-1.191404	3.215862	-0.515072
14	1	0	-0.887105	2.471902	1.058952
15	8	0	0.187117	-1.785781	-0.324240
16	1	0	-0.018112	-0.758605	1.932707
17	1	0	1.159764	-1.801738	-0.528092
18	8	0	2.838376	-1.753365	-0.777662
19	1	0	3.151791	-1.118891	-0.101571
20	1	0	3.026342	-1.340359	-1.626836
21	8	0	3.112495	0.089783	1.251706

22	1	0	2.154224	0.084979	1.429562
23	1	0	3.556107	-0.009571	2.099130

4 water Intermediate 1

Center Number	Atomic Number	Atomic Type	Coordinates (Angstroms) X	Y	Z
1	6	0	2.987743	-0.323037	-0.006513
2	9	0	3.609220	0.580307	0.772089
3	9	0	3.562095	-0.289771	-1.219954
4	9	0	3.189869	-1.537913	0.522695
5	6	0	1.463342	-0.049397	-0.141819
6	8	0	0.793987	-0.839933	-0.763688
7	16	0	0.914696	1.420836	0.639009
8	6	0	-0.861375	1.370855	0.163086
9	1	0	-1.347486	2.029311	0.883449
10	1	0	-1.207367	0.359880	0.358970
11	6	0	-1.111381	1.809762	-1.272780
12	1	0	-2.179411	1.734113	-1.488281
13	1	0	-0.788189	2.840259	-1.434416
14	1	0	-0.585475	1.165117	-1.978404
15	8	0	-4.095161	0.597430	1.076694
16	1	0	-4.972988	0.859605	1.366067
17	1	0	-4.166996	0.363423	0.122369
18	8	0	-3.862180	-0.223437	-1.498823
19	1	0	-3.139010	-0.889651	-1.374488
20	1	0	-4.509579	-0.629350	-2.081686
21	8	0	-1.919641	-1.946012	-0.823036
22	1	0	-1.002740	-1.684015	-0.988415
23	1	0	-2.011031	-1.938521	0.150228
24	8	0	-2.327028	-1.340927	1.849540
25	1	0	-3.035489	-0.667071	1.768406
26	1	0	-2.529819	-1.876156	2.621239

4 water Transition state

Center Number	Atomic Number	Atomic Type	Coordinates (Angstroms) X	Y	Z
1	6	0	1.792647	-1.163021	-0.122403
2	9	0	2.189828	-1.584520	1.092405
3	9	0	2.773695	-0.410424	-0.647049
4	9	0	1.642769	-2.244404	-0.903766
5	6	0	0.476563	-0.343555	-0.044241
6	8	0	-0.075159	-0.081033	-1.227903

7	16	0	0.638296	1.011383	1.204894
8	6	0	0.463967	2.495627	0.130622
9	1	0	0.423637	3.302457	0.867201
10	1	0	-0.503153	2.460896	-0.365517
11	6	0	1.607139	2.701947	-0.852788
12	1	0	1.461801	3.646734	-1.385869
13	1	0	2.569361	2.739202	-0.338698
14	1	0	1.636540	1.898505	-1.588409
15	8	0	-0.565357	-1.396009	0.393904
16	1	0	-0.810687	-1.071808	-0.728334
17	1	0	-1.261038	-0.963598	0.974651
18	8	0	-2.632590	-0.134613	1.474063
19	1	0	-2.620179	0.558880	0.760805
20	1	0	-2.536370	0.322219	2.316035
21	8	0	-2.569565	1.209709	-0.828119
22	1	0	-1.715575	0.913586	-1.197024
23	1	0	-3.238170	0.620186	-1.212639
24	8	0	-4.173120	-1.085314	-0.720403
25	1	0	-4.029972	-1.094737	0.237247
26	1	0	-4.961125	-1.608410	-0.891545

4 water Intermediate 2

Center Number	Atomic Number	Atomic Type	Coordinates (Angstroms) X	Y	Z
1	6	0	-1.601512	-0.859949	-0.136142
2	9	0	-2.179159	-1.005658	-1.335005
3	9	0	-2.397393	-0.087721	0.620301
4	9	0	-1.559670	-2.080565	0.450140
5	6	0	-0.163306	-0.278235	-0.251215
6	8	0	0.377441	-0.134664	1.041587
7	16	0	-0.184543	1.368146	-1.110584
8	6	0	-0.091474	2.622650	0.231928
9	1	0	0.064391	3.537666	-0.346770
10	1	0	0.817572	2.452065	0.805339
11	6	0	-1.315424	2.751743	1.126900
12	1	0	-1.188575	3.612418	1.791587
13	1	0	-2.222979	2.902272	0.539331
14	1	0	-1.448742	1.863073	1.741655
15	8	0	0.521720	-1.198872	-1.021768
16	1	0	0.592813	-1.041177	1.365018
17	1	0	1.424754	-0.847799	-1.240255
18	8	0	3.029736	-0.314025	-1.424684
19	1	0	3.207453	0.192459	-0.604802
20	1	0	3.101058	0.320721	-2.144895
21	8	0	2.940856	0.966287	0.993001
22	1	0	2.030704	0.652002	1.162535
23	1	0	3.430993	0.871619	1.814559

24	8	0	1.174498	-2.748893	1.361631
25	1	0	2.109336	-2.972777	1.413253
26	1	0	0.936177	-2.843298	0.426901

5 water Intermediate 1

Center Number	Atomic Number	Atomic Type	Coordinates (Angstroms) X	Y	Z
1	6	0	2.035049	-0.442281	-0.882089
2	9	0	2.616043	-0.957072	0.211020
3	9	0	2.946367	0.362988	-1.483902
4	9	0	1.732331	-1.420010	-1.726367
5	6	0	0.791358	0.421989	-0.539601
6	8	0	-0.101819	0.550023	-1.333966
7	16	0	0.932105	1.315818	0.986477
8	6	0	-0.684337	2.193406	0.962154
9	1	0	-0.844079	2.466234	2.007114
10	1	0	-1.446024	1.466873	0.679757
11	6	0	-0.696222	3.416023	0.054313
12	1	0	-1.675258	3.899993	0.115885
13	1	0	0.064271	4.141932	0.349341
14	1	0	-0.526037	3.131403	-0.983989
15	8	0	-0.145317	-1.935252	0.545580
16	1	0	-1.105787	-2.063922	0.595609
17	1	0	0.180556	-2.042013	1.451554
18	8	0	-3.625577	0.526975	0.035423
19	1	0	-3.289152	0.256613	-0.852925
20	1	0	-4.463434	0.974424	-0.113324
21	8	0	-2.511831	-0.730928	-2.080772
22	1	0	-1.585572	-0.446069	-2.092361
23	1	0	-2.510758	-1.575420	-1.608047
24	8	0	-2.997304	-2.112006	0.465760
25	1	0	-3.402229	-1.226592	0.567050
26	1	0	-3.576274	-2.746775	0.896375
27	8	0	1.024431	-1.409829	3.105719
28	1	0	1.118334	-0.522337	2.732003
29	1	0	0.679342	-1.287284	3.994946

5 water Transition state

Center Number	Atomic Number	Atomic Type	Coordinates (Angstroms) X	Y	Z
1	6	0	2.203546	-1.185493	-0.306107
2	9	0	2.594695	-1.775252	0.838822
3	9	0	3.203592	-0.399302	-0.736694

4	9	0	2.015520	-2.148910	-1.221714
5	6	0	0.914437	-0.344275	-0.107331
6	8	0	0.358155	0.087361	-1.234066
7	16	0	1.129429	0.825227	1.311075
8	6	0	0.941920	2.441567	0.450558
9	1	0	0.905374	3.141535	1.289537
10	1	0	-0.029240	2.466597	-0.037621
11	6	0	2.077367	2.780128	-0.504236
12	1	0	1.928091	3.788310	-0.903432
13	1	0	3.043972	2.748774	0.002111
14	1	0	2.100885	2.084194	-1.342635
15	8	0	-0.160160	-1.422689	0.202227
16	1	0	-0.403711	-0.935679	-0.857355
17	1	0	-0.827604	-1.052367	0.853239
18	8	0	-2.139545	-0.223038	1.512686
19	1	0	-2.159598	0.486207	0.798364
20	1	0	-1.874055	0.216497	2.328374
21	8	0	-2.134551	1.355668	-0.572174
22	1	0	-1.350135	1.096810	-1.085650
23	1	0	-2.932411	1.103970	-1.087046
24	8	0	-4.633440	-1.216907	0.727248
25	1	0	-3.802704	-0.982658	1.183566
26	1	0	-4.702092	-2.175498	0.760082
27	8	0	-4.441674	0.304629	-1.561913
28	1	0	-5.250003	0.775992	-1.781894
29	1	0	-4.655802	-0.286882	-0.809929

5 water Intermediate 2

Center Number	Atomic Number	Atomic Type	Coordinates (Angstroms) X	Y	Z
1	6	0	1.226796	-1.541387	0.180139
2	9	0	1.723926	-1.956671	1.350381
3	9	0	2.252494	-1.347920	-0.664112
4	9	0	0.487564	-2.561313	-0.332827
5	6	0	0.347163	-0.267639	0.330228
6	8	0	-0.141881	0.102165	-0.924901
7	16	0	1.312005	1.144687	1.062233
8	6	0	1.878639	2.153071	-0.368282
9	1	0	2.129554	3.099682	0.118363
10	1	0	1.024901	2.344958	-1.015118
11	6	0	3.070496	1.613142	-1.146362
12	1	0	3.409258	2.368251	-1.863220
13	1	0	3.902300	1.375334	-0.480118
14	1	0	2.807883	0.713182	-1.699872
15	8	0	-0.637891	-0.642192	1.224358
16	1	0	-0.820396	-0.574575	-1.219144
17	1	0	-1.241687	0.113610	1.378694

18	8	0	-2.574669	1.364811	1.361240
19	1	0	-2.312582	1.917723	0.582109
20	1	0	-2.631494	1.947786	2.124422
21	8	0	-1.686981	2.386450	-0.932669
22	1	0	-1.072680	1.632602	-1.062465
23	1	0	-2.276486	2.388485	-1.693508
24	8	0	-4.106886	-0.436141	-0.099613
25	1	0	-3.732253	0.163739	0.573160
26	1	0	-4.874835	-0.857162	0.296125
27	8	0	-2.089063	-1.551843	-1.607468
28	1	0	-1.855456	-2.425734	-1.277059
29	1	0	-2.863569	-1.258852	-1.080995
